# Predictive Model for *Listeria monocytogenes* in RTE Meats Using Exclusive Food Matrix Data

**DOI:** 10.3390/foods13233948

**Published:** 2024-12-06

**Authors:** N. A. Nanje Gowda, Manjari Singh, Gijs Lommerse, Saurabh Kumar, Eelco Heintz, Jeyamkondan Subbiah

**Affiliations:** 1 Department of Food Science, University of Arkansas Division of Agriculture, Fayetteville, AR 72204, USA; 2 Food Preservation and Protection, Kerry Taste & Nutrition, 6708 Wageningen, The Netherlands; 3 Food Preservation and Protection, Kerry Taste & Nutrition, Beloit, WI 53511, USA

**Keywords:** gamma model, RTE meat, lag phase duration, clean label, organic acids

## Abstract

Post-processing contamination of *Listeria monocytogenes* has remained a major concern for the safety of ready-to-eat (RTE) meat products that are not reheated before consumption. Mathematical models are rapid and cost-effective tools to predict pathogen behavior, product shelf life, and safety. The objective of this study was to develop and validate a comprehensive model to predict the *Listeria* growth rate in RTE meat products as a function of temperature, pH, water activity, nitrite, acetic, lactic, and propionic acids. The *Listeria* growth data in RTE food matrices, including RTE beef, pork, and poultry products (731 data sets), were collected from the literature and databases like ComBase. The growth parameters were estimated using the logistic-with-delay primary model. The good-quality growth rate data (*n* = 596, R^2^ > 0.9) were randomly divided into 80% training (*n* = 480) and 20% testing (*n* = 116) datasets. The training growth rates were used to develop a secondary gamma model, followed by validation in testing data. The growth model’s performance was evaluated by comparing the predicted and observed growth rates. The goodness-of-fit parameter of the secondary model includes R^2^ of 0.86 and RMSE of 0.06 (μ_max_) during the development stage. During validation, the gamma model with interaction included an RMSE of 0.074 (μ_max_), bias, and accuracy factor of 0.95 and 1.50, respectively. Overall, about 81.03% of the relative errors (RE) of the model’s predictions were within the acceptable simulation zone (RE ± 0.5 log CFU/h). In lag time model validation, predictions were 7% fail-dangerously biased, and the accuracy factor of 2.23 indicated that the lag time prediction is challenging. The model may be used to quantify the *Listeria* growth in naturally contaminated RTE meats. This model may be helpful in formulations, shelf-life assessment, and decision-making for the safety of RTE meat products.

## 1. Introduction

*Listeria monocytogenes* is a gram-positive, facultative pathogen that can cause severe foodborne illness in humans. It has been a significant threat to the safety of ready-to-eat (RTE) meats that are frequently implicated in *listeriosis* outbreaks [1,2]. *Listeria* is ubiquitous and can grow in a range of temperature (−1.5 to 45 °C), pH (4.3 to 9.4), aw (>0.92), and NaCl concentration up to 13%. Prevention of post-processing contamination in RTE food during handling, processing, and packaging facilities has remained a challenge over the years [3,4]. Ready-to-eat meals (31%), cooked meat and seafood (16.80%), dairy & poultry products (14.47%), and fruits and vegetables (2.2–15.6%) are the leading food vehicles for *Listeria* contamination [5,6,7]. According to the EU 2022 report, the occurrence of *Listeria* in RTE food products (3.5–5.4%) includes pork meat products (2.7%), sausages (2.5–3.1%), poultry meat products (broilers and turkeys—1.3%), bovine meat products (3.9%), fish and fishery products (3.5–5.4%), soft and hard cheeses (0.69%), milk (0.3%), and fruits, vegetables, and juices (3.0%) [8]. The USDA-FSIS initiated a “zero tolerance” policy (≤1 CFU in 25 g sample) for RTE food products [9]. In contrast, contamination <100 CFU/g at the time of consumption was imposed by EU Regulation (EC) 2073/2005 [10]. *Listeria* tolerance as per EC regulation is also followed by [11], Australia, and New Zealand [12]. These stringent food safety policies have increased pressure on food industries to control post-process contamination in RTE meat, seafood, and poultry products. In addition, the economic impact of *Listeria* on food industries is devastating due to the costs associated with recalls and loss of productivity. *Listeriosis* economic burden in the United States alone was estimated to be between 2.3 to 22 billion USD per year, while the global burden has never been accurately estimated [13].

RTE products consumed without reheating, such as deli meat and frankfurters, are considered high-risk products [14,15]. *Listeria* can be resistant to preservation methods and can grow even under refrigerated temperatures, which is a major concern for the food industry to ensure microbial safety. Therefore, post-processing hurdle techniques are required to inactivate or inhibit the growth of *Listeria* in RTE meats throughout the storage period. Post-packaging treatments such as irradiation and high-pressure processing approaches suffer from limitations related to low consumer acceptability, throughput, and high capital costs [16]. Therefore, the use of organic acids as antimicrobials is one of the most effective methods to inhibit *Listeria* growth and enhance the shelf life of RTE meats. In addition, approval of organic acids as food preservatives [17] and their recognition as a “generally recognized as safe” (GRAS) compound by FDA-21.CFR [18] has increased their usage of RTE meats. Acetates, lactates, and nitrites are the most preferred antimicrobial compounds in meat, poultry, and seafood [19,20].

The challenge studies to assess the effect of antimicrobials on microbial kinetics and product shelf life are expensive, impractical to perform during frequent changes in product recipes, and not ideal for large-scale sampling [21]. In this context, predictive modeling is a rapid solution to simulate the effect of change in product formulation on product safety and shelf life of products. The models could be built with minimal microbial studies and used to predict *Listeria* behavior. This can reduce a significant amount of challenging studies, saving time and resources through quick simulation. In addition, mathematical models can also quantify microbial behavior as a function of various environmental and inhibiting substances. The use of gamma concept modeling has gained significant interest. These models can simulate the combined effects of multiple factors on pathogen behavior and help in risk assessment [22,23]. The European Regulation [10] also suggested food business operators may use predictive models as a tool for evaluating compliance to microbiological criteria throughout the shelf-life of RTE foods. The existing broth models in the literature are often developed on liquid media, which offer highly conservative predictions due to the high mobility of nutrients and favorable growth conditions. These models fail to account for the complex interactions within real meat matrices. Additionally, the predictions may not represent real-world conditions resulting in over processing of foods. Therefore, the development and validation of an improved model considering the synergistic effect of environmental and antimicrobial factors in real meat matrices instead of conventional liquid growth media is crucial. Considering the combined effects of clean label solutions and environmental factors provides a more practical and reliable predictive tool to enhance food safety and precisely optimize preservative usage in RTE meat products.

Over the last decade, several secondary models for *Listeria* growth prediction as a function of several factors have been developed [23,24,25,26,27,28,29,30,31,32]. Most of these models were developed with lab culture media and validated either in liquid media alone and/or a combination of food matrices and culture media [33,34]. The broth models can only provide useful insights into microbial growth behavior and aid in predicting growth in food matrices [35]. Broth models are often considered to overestimate the growth rate due to the high mobility of nutrients in culture media and contain a range of compounds that favor rapid microbial growth [21,36]. This may result in over-prediction of growth rates when applied to food, which may not be acceptable for food quality [37,38]. These models often predict the worst-case scenario and are still models of real food. It is more effective to develop the model on real food matrices and optimize the use of preservatives. This is particularly important to reduce the overuse of organic acids as antimicrobials, as they can affect the sensory properties of RTE meat. In contrast, the food matrix is more complex due to pH, a_w_, low mobility of nutrients, chemical composition, and background microflora characteristics [36,39]. As a result, lab broth models are inappropriate and may not accurately predict the *Listeria* behavior in the food matrix [40,41]. A literature survey suggests that very limited *Listeria* models have been developed and validated in real food matrices [26,42,43]. Predictive models are yet to be developed in RTE meat foods and validated in RTE meat foods matrices to describe the combined effect of sodium nitrite and organic acids with other factors such as temperature, pH, and a_w_/NaCl, etc. The literature search revealed that only one study [43] reported a gamma-type model that was developed and validated in combined broth and RTE meat data as a function of acetic and propionic acids. Therefore, the objective of this study was to develop and validate a comprehensive *Listeria* growth model using exclusively RTE food matrices data to describe the individual and synergistic effects of various factors.

## 2. Materials and Methods

### 2.1. Data Collection and Growth Rate Estimation

*Listeria* growth curves (*n* = 731) in different RTE food matrices (beef, pork, poultry) were collected (shown in Table 1, Table 2 and Table 3) from previous publications, ComBase, and data directly contributed by authors through personal communication. Raw data were always extracted, when raw data were not available the published graphs were scanned and individual points were digitalized using plot digitizer 2.6.11 (https://sourceforge.net/projects/plotdigitizer/files/) software (accessed on 8 October 2022), following the method [44]. The logistic-with-delay primary model (Equation (1)) was fitted to growth curves to determine the exponential growth rate (µ_max_) and lag time (t_lag_) [45]. The non-linear fitting was performed (*lsqcurvefit* function, Optimization Toolbox) using MATLAB R2022b (The MathWorks, Natick, MA, USA).

(1) log ⁡Nt=log⁡N0t<tlag log⁡Nt=log⁡Nmax1+NmaxN0−1∗ exp−μmax(t−tlag)t≥tlag

where t is time (h), N_t_ is cell concentration (CFU/g) at time t, N_0_ is the initial cell concentration (CFU/g), N_max_ is maximum population density (CFU/g), μ_max_ is the maximum exponential growth rate (h^−1^), and t_lag_ is the lag time (h).

If the maximum exponential growth rates were directly taken from publications with different primary models, such data were transformed. The growth rates were transformed from the Gompertz, Logistic, and Baranyi models to the logistic-with-delay model using conversion factors of 0.84, 0.86, and 0.97, respectively. Similarly, lag times were transformed from Gompertz, Baranyi, and Logistic by using 0.82, 0.97, and 0.95, respectively [46]. The environmental factors considered in the study were temperature, pH, water activity, sodium nitrite, acetic acid, lactic acid, and propionic acid. The collected data included *Listeria* growth in RTE beef, pork, and poultry meat products.

### 2.2. Data Selection Criteria and Assumptions

The maximum exponential growth rates obtained from 731 curves were filtered based on quality criteria (reported in Table 1) to select good-quality data for the model development. After filtering, a total of 596 datasets were selected for model development and validation. The selected data included variables such as temperature (−1.5 to 37 °C), pH (4.8 to 6.8), water activity (0.9 to 0.997), nitrites (0 to 8.48 mM), acetic acid (0 to 8.95 mM), lactic acid (0 to 3.30 mM), and propionic acid (0 to 2.12 mM). The selected dataset was randomly divided into a model development dataset (*n* = 480) and a validation dataset (*n* = 116). The selected *Listeria* data includes 20 beef products, 30 pork products, and 22 poultry products from about 76 different publications (Table 1, Table 2 and Table 3).

When no information was available in the publications, pH was assumed based on similar types of meat products reported in other studies. If the formulation included nitrite but no concentration was reported, an average nitrite of 98.5 ppm for all other meat products was assumed as suggested by [49]. The water activity (a_w_) values were recalculated from NaCl concentration using the formula [50] shown in Equations (2) and (3). The missing concentration of NaCl (%) was calculated from the molar equivalent of sodium in the products obtained from the food databank of DTU Food databank [51] or the USDA food composition table [52], as suggested earlier [53]. The undissociated organic acid concentrations (mM) in water phase salts were calculated (Equation (4)), as only undissociated acids exhibit antimicrobial activity, followed by anions activity to a lesser extent [54].

a_w_ = 1 − 0.0052471 ∗ %WPS − 0.00012206 ∗ %WPS^2^ (2)

where, WPS refers to water phase salts, which can be calculated by the following:

(3) $$WPS=\frac{\left(\%NaCl\;or\;acid\;*\;100\right)}{\left(100-\%dry\;matter+\%NaCl\;or\;acid\right)}$$

(4) $$Undissociated\;acid,\;OA\;\left(mM\right)=\frac{Total\;acid\;(mM)}{1+10^{pH-pka}}$$

where,

(5) $$\mathrm{Total}\;\mathrm{acid}\;(\mathrm{mM})=\frac{\left(\frac{\%\;\mathrm{WPS}}{\mathrm{Molar}\;\mathrm{mass}\;\mathrm{of}\;\mathrm{organic}\;\mathrm{acid}\;\mathrm{salt}\;\left(\;\frac{g}{\mathrm{mol}}\right)}\right)}{\left(\frac{\mathrm{Mass}\;\mathrm{of}\;\mathrm{water}\;+\;\mathrm{organic}\;\mathrm{acid}\;\mathrm{salt}\;\;\left(g\right)}{\mathrm{Mass}\;\mathrm{of}\;\mathrm{water}\;\mathrm{in}\;\mathrm{which}\;\mathrm{organic}\;\mathrm{acid}\;\mathrm{salt}\;\mathrm{was}\;\mathrm{dissolved}\;\;\left(g\right)\;}\right)}*\;1000$$

The pka is the disassociation constant determining an acid’s strength, and the values of pka for different organic acids reported in Table 2 were used.

### 2.3. Development of Secondary Growth Rate Model

The gamma concept model was used to describe the influence of the environment and inhibiting conditions on microbial growth. The square root transformation for μ_max_ values was applied to homogenize the variance as suggested earlier [28,30,55]. Modeling of maximum exponential growth rates (μ_max_, h^−1^) as a function of temperature, pH, water activity, nitrite, and organic acids was performed using Equation (6).

(6) $$\sqrt{{µ}_{max}}=\sqrt{{µ}_{opt}\cdot \gamma \left(T\right)\cdot \gamma \left(pH\right)\cdot \gamma \left(a_{w}\right)\cdot \gamma \left(NIT\right)\cdot \gamma \left(OA\right)\cdot \xi \left(T,\;pH,\;a_{w},\;NIT,\;OA\right)}$$

Here, µ_max_ is the maximum exponential growth rate (log CFU/g/h), and the gamma terms γ(T), γ(pH), γ(a_w_), γ(NIT), and γ([OA]) refer to the effects of temperature, pH, water activity, nitrite, and organic acids, respectively. Xi (ξ) is the quantitative effect of interactions between the parameters. The gamma model was used to describe the effect of temperature (Equation (7)), pH (Equation (8)), and water activity (Equation (9)) on the growth rate [22,25].

(7) $$\gamma (T)=\left\{\begin{array}{c} 0\;\;\;\;\;\;\;\;\;\;\;\;\;\;\;\;\;\;\;\;\;\;\;\;\;\;\;\;\;\;\;\;\;\;\;\;\;\;\;\;\;,\;T\leq T_{min}\;\\ {\left(\frac{\left(T-T_{min}\right)}{\left(T_{opt}-T_{min}\right)}\right)}^{2}{\;\;\;,T}_{min}<T<T_{opt}\\ 0\;\;\;\;\;\;\;\;\;\;\;\;\;\;\;\;\;\;\;\;\;\;\;\;\;\;\;\;\;\;\;\;\;\;\;\;\;\;\;\;\;,\;T\geq T_{opt} \end{array}\right.$$

(8) $$\gamma (\mathrm{pH})=\left\{\begin{array}{c} \;\;0\;\;\;\;\;\;\;\;\;\;\;\;\;\;\;\;\;\;\;\;\;\;\;\;\;\;\;\;\;\;\;\;\;\;\;\;\;\;\;\;\;\;\;\;\;\;\;\;\;\;\;\;\;\;\;\;\;\;\;\;\;\;\;\;\;\;\;\;\;\;\;\;\;\;\;,\;pH\leq {pH}_{min}\;\\ \frac{\left(pH-{pH}_{min}\right)*\left({pH}_{max}-pH\right)}{\left({pH}_{opt}-{pH}_{min}\right)\left({pH}_{max}-{pH}_{opt}\right)},\;\;\;{pH}_{min}<pH<{pH}_{max}\\ \;\;0\;\;\;\;\;\;\;\;\;\;\;\;\;\;\;\;\;\;\;\;\;\;\;\;\;\;\;\;\;\;\;\;\;\;\;\;\;\;\;\;\;\;\;\;\;\;\;\;\;\;\;\;\;\;\;\;\;\;\;\;\;\;\;\;\;\;\;\;\;\;\;\;\;\;\;,\;pH\geq {pH}_{max} \end{array}\right.$$

(9) $$\gamma (a_{w})=\left\{\begin{array}{c} 0,\;\;\;\;\;\;\;\;\;\;\;\;\;\;\;\;\;\;\;\;\;\;\;\;\;\;\;\;\;\;\;\;\;\;\;\;\;\;\;,a_{w}<a_{wmin}\\ \left(\frac{{(a}_{w}-a_{wmin})}{\left(a_{wopt}-a_{wmin}\right)}\right)\;\;\;\;{,a_{w}>a}_{wmin}<a_{wopt} \end{array}\right.$$

where T_opt_, pH_opt_, T_min_, pH_min_, a_wmin_, and pH_max_ are theoretical minimal, optimal, and maximal values of temperature, pH, and water activity, respectively, for *Listeria* growth. The inhibitory effects of undissociated nitrite (Equation (10)), undissociated lactate (Equation (11)), undissociated acetate (Equation (12)) and propionate (Equation (12)), were modeled [47] as follows:

(10) $$\gamma \left(Nit\right)=\left\{\begin{array}{c} 1-\frac{NIT}{{MIC}_{Nit}}\;\;\;\;Nit<{MIC}_{Nit}\\ 0\;\;\;\;\;\;\;\;\;\;\;\;\;\;\;\;\;\;\;\;\;\;Nit\;\geq {MIC}_{Nit} \end{array}\right.$$

(11) $$\gamma \left(\left[{OA}_{1}\right]\right)=\left\{\begin{array}{c} 1-\frac{\left[{OA}_{1}\right]}{{MIC}_{u}}\;\;\;\;[{OA}_{1}]<{MIC}_{u}\\ 0\;\;\;\;\;\;\;\;\;\;\;\;\;\;\;\;\;\;\;\;[{OA}_{1}]\;\geq {MIC}_{u} \end{array}\right.$$

(12) $$\gamma \left({[OA}_{2\;or\;3}]\right)=\left\{\begin{array}{c} 1-\sqrt{\frac{\left[{OA}_{2\;or\;3}\right]}{{MIC}_{U}}}\;\;\;\;[{OA}_{2\;or\;3}]<{MIC}_{U}\\ 0\;\;\;\;\;\;\;\;\;\;\;\;\;\;\;\;\;\;\;\;\;\;\;\;\;\;\;\;[{OA}_{2\;or\;3}]\;\geq {MIC}_{U} \end{array}\right.$$

where MIC_Nit_ and MIC_U_ are the minimal inhibitory concentrations of nitrite and organic acids, respectively; Nit and [OA] are undissociated nitrite (mM) and respective organic acid (mM) concentrations calculated using Equation (4), respectively. The initial guess values for MICs of nitrite and organic acids as shown in Table 2 were used.

An approach by [47] was used to model the interaction effect (Xi) between gamma factors using Equations (13)–(19).

(13) $$\xi =\left\{\begin{array}{c} 1,\;\psi \leq 0.5\\ 2(1-\psi )\\ 0,\;\psi \geq 1\; \end{array}\right.,0.5<\psi <1$$

(14) $$\psi =\sum_{i}\frac{\phi_{i}}{2\underset{j\neq 1}{\prod }\left(1-\phi_{j}\right)}$$

(15) $$\phi (T)={\left(1-\sqrt{\gamma (T)}\right)}^{3}$$

(16) $$\phi (\mathrm{pH})={\left(1-\sqrt{\gamma (pH)}\right)}^{3}$$

(17) $$\phi (a_{w})={\left(1-\sqrt{\gamma (a_{w})}\right)}^{2}$$

(18) $$\phi (\mathrm{NIT})={\left(1-\gamma (Nit)\right)}^{2}\;$$

(19) $$\phi ([\mathrm{OA}])={\left(1-(\left[{OA}_{1}\right])\cdot \left({[OA}_{2}]\right)\cdot ({[OA}_{3}])\right)}^{2}$$

where [OA_1_], [OA_2_], and [OA_3_] are the undissociated lactic, acetic, and propionic acids, respectively. The Xi (ξ) value indicates the growth or no growth boundary, and the value varies between 0 and 1. The Xi is calculated from Psi (ψ); Psi < 0.5 indicates no interaction (ξ = 1); if Psi is >1, no growth occurs (ξ = 0), and if Psi is <1 and >0.5, μ_max_ (h^−1^) is reduced depending on Psi values.

### 2.4. Secondary Modeling Approach

The development of the secondary model involves the estimation of gamma parameter values using the model development dataset (*n* = 480) in initial fitting procedures. Two studies [28,43] have demonstrated that both “sequential” and “simultaneous” modeling approaches can be used to determine the gamma parameters. The variables estimated by “simultaneous” modeling had no strong correlation in the matrix of correlation analysis, and these parameters were used to develop the final model [43]. The sequential modeling approach involves the estimation of temperature parameters first using temperature variable data only, followed by pH parameters using pH variable data, and so on. In contrast, the simultaneous approach involves the estimation of all parameters at once using a complete dataset. In the current study, due to limited food matrices datasets, only a “simultaneous” modeling approach was employed to build the secondary model. This method generally offers better fit and better parameter estimates in limited and disparate datasets and offers a time advantage [43]. The Mathworks (MATLAB, R2022b Update2) with Optimization toolbox was used for the secondary modeling component of the study. The “lsqcurvefit” function was used to compute the minimum sum of squares of the residual errors by the non-linear fitting module. The “nlparci” function was used to estimate the confidence intervals of the parameters by linear approximation [29].

### 2.5. Secondary Growth Rate Model Validation

The developed model was validated using the validation dataset (*n* = 116). The predicted zero growth rates were replaced by a small value of 0.0003 h^−1^ to estimate numerical values for bias and accuracy validation criteria, as suggested earlier [56]. During model development, the goodness of fit was evaluated using the coefficient of determination (R^2^) and root mean square error (RMSE). Followed by the validation of the developed model using RMSE, bias factor (B_f_), accuracy factor (A_f_), percent bias (% B), and percent discrepancy (% D) using Equations (20)–(24), respectively. The proportion of relative errors (RE) falling within the acceptable simulation zone (ASZ) with an acceptable boundary of ± 0.5 RE was also used to evaluate the model performance during validation [34,57]. It is suggested that a model describing the growth kinetics of *Listeria* is considered as good if the B_f_ is between 0.9 and 1.05, acceptable if it ranges from 0.7 to 0.9 or 1.06 to 1.15, and unacceptable if it is less than 0.7 or greater than 1.15 [58]. An accuracy factor between 1 and 1.5 is deemed acceptable, while an accuracy factor greater than 1.5 is unacceptable.

(20) $$R^{2}=1-\frac{{\Sigma_{i}\left(y_{i}-\hat{y}i\right)}^{2}}{{\Sigma_{i}\left(y_{i}-\bar{y}\right)}^{2}}$$

(21) $$B_{f}=10^{\sqrt{\left(\sum log({µ}_{max}predicted/{µ}_{max}observed\right)/n)}}$$

(22) $$A_{f}=10^{\sqrt{\left(\sum |log({µ}_{max}predicted/{µ}_{max}observed\right)|/n)}}$$

%D = (A_f_ − 1) × 100% (23)

%B = sgn (ln B_f_) ∗ (exp (abs ((ln B_f_)−1)) × 100% (24)

### 2.6. Development of Lag Time Model and Its Validation

**Figure 1:**
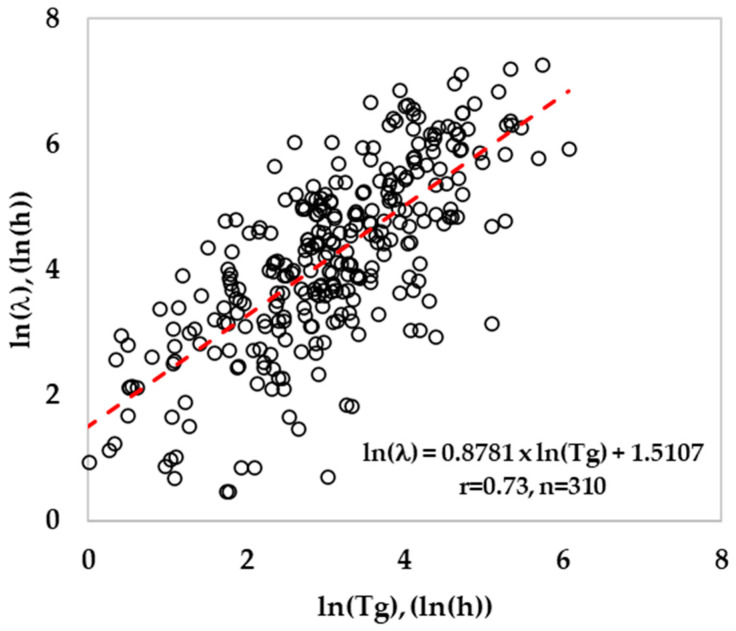
Relationship between generation time and lag time for *Listeria* (training data).

The lag time was modeled by following the relative lag time concept (RLT) by estimating the parameter K [25]. This parameter explains the physiological state of cells, a constant value for cells with similar pre-inoculation history. When these cells are exposed to the same favorable growth conditions, the amount of work to be done by bacteria will remain constant to adapt to the newly growing environment. Therefore, the ratio of ln(l/Tg) will produce a constant value, and the linear regression analysis (Figure 1) between ln(l) and ln(Tg) should produce a slope value close to 1. The median value for ln(l/Tg) ratios was estimated, and the exp(median) is the parameter K. Finally, the lag time was calculated using Equation (25). The validation of lag time prediction was done by estimating the bias and accuracy factors between observed and predicted ln(l) using Equations (21) and (22). The mean prediction error (MPE) for lag time was calculated using Equation (26).

(25) $$\lambda =K*Tg=K*\left(\frac{ln(2)}{\mu_{max}}\right)$$

(26) $$MPE\left(\%\right)=\frac{\sum \frac{\left|(obs.\lambda )\;-\;(pred.\lambda )\right|}{\left(obs.\lambda \right)}}{n}*100$$

## 3. Results

### 3.1. Development of a Secondary Model for Listeria Growth Prediction

**Figure 2:**
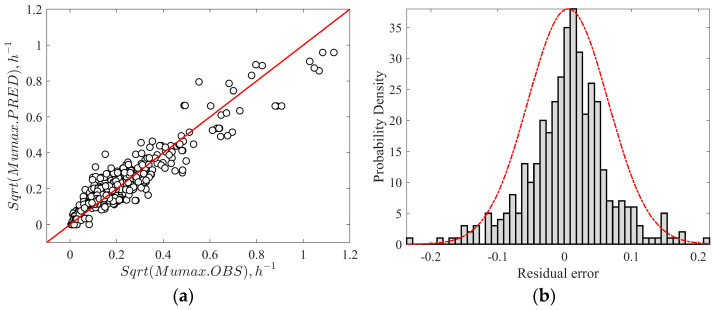
(**a**) Correlation between the observed and predicted growth rates of *L. monocytogenes*. (**b**) Distribution of the relative error of predicted values by the secondary model.

The logistic-with-delay primary model was fitted to estimate the maximum exponential growth rates (µ_max_, log CFU/h) and used for secondary modeling. The logistic-with-delay model was used due to widespread usage and recognition in the literature as one of the most accurate models to describe the sigmoidal growth curve of *Listeria*. This model also accounts for the lag phase accurately, which enhances its reliability and usage. Similarly, the gamma secondary equation was employed in the current study due to its simplicity, robustness, and ability to quantify the effect of multiple factors on the growth of *Listeria* using the gamma function. Additionally, the gamma equations also describe the growth and no-growth boundaries through a graphical representation. In this context, a secondary model was developed by fitting Equation (6) to growth rates as a function of seven environmental factors. To be consistent with the literature, optimum growth rate (μ_opt_) is reported as h^−1^, while the actual unit of μ_opt_ is log CFU/h. During the initial fitting of gamma equations to the raining dataset, due to the limited growth rate, the pH_opt_ and pH_max_ values were not estimated. Instead, these values were selected [25,59] and fixed at 7 (pH_opt_), and 9.6 (pH_max_), respectively, in the simultaneous fitting procedure. Except for fixed parameters, the remaining parameters were simultaneously estimated by following the earlier procedures [28,32]. The optimal growth rate (µ_opt_) of the secondary model (Equation (6)) estimated by the model in the present study was 1.126 h^−1^ across all meats, which was within the range of literature values for processed meat (Table 3). The confidence interval for optimal growth rate (µ_opt_) was slightly high (0.65 to 1.66 h^−1^) due to the variability in growth rates across the meat products and strain differences. The large variability causes difficulties in defining the growth trends (using µ_opt_) in a mixture of food products. Similarly, a study [29] reported a high variability of µ_opt_ between 0.18 and 2.02 h^−1^ with a mean value of 0.49 ± 0.32 h^−1^, which includes meat (0.63 ± 0.73 h^−1^), seafood (3.61 ± 5.71 h^−1^), beef (0.18 ± 0.14 h^−1^), and poultry (0.765 ± 0.83 h^−1^) products. Figure 2a represents the correlation between the observed and predicted growth rates in meat products by the secondary model. Figure 2b shows the histogram plot of residual error, which is an indicator of over- and under-predictions of growth rates by the model. The negative values refer to over-predictions, the positive values refer to under-predictions, and values closer to the zero-region column refer to accurate predictions. The histogram plot with a symmetrical distribution of residual error around zero demonstrates the great robustness and accuracy of the model. In the development stage, the secondary model had a coefficient of determination R^2^ of 0.86 and RMSE of 0.06 μ_max_, demonstrating the overall quality of fit across the seven environmental conditions. On the other hand, the gamma model developed for *Listeria* growth prediction in meat products had an RMSE of 0.081 and R^2^ of 0.63 [22]. In another study, RMSE ranged between 0.919 and 1.148 and R^2^ adj from 0.81 to 0.88 for Ratkowsky square root model fitting in *Listeria* growth rate data in high-pressure processed cooked ham [31].

#### 3.1.1. Estimation of Gamma Model Parameters

**Figure 3:**
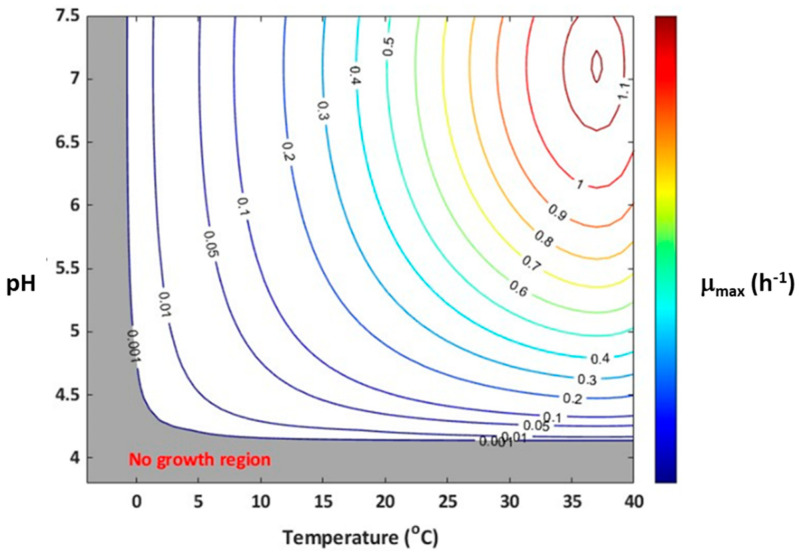
Growth/no growth contour plot of µ_max_ as a function of pH and temperature. The contour lines represent µ_max_ predicted using Equation (6) with the model parameters reported in Table 3.

The effect of minimum and maximum temperature on the growth rate of *Listeria* was estimated and summarized in Table 3. The sequential fitting of gamma equations to individual datasets could not be performed due to the limited data available for each factor. Therefore, the model parameters were simultaneously estimated at once using the whole data, following the method reported earlier [28,43]. The temperature parameters, including T_opt_ of 37.0 °C and T_min_ of −1.57 °C, were estimated in this study. The estimated T_min_ and T_opt_ values for *Listeria* were within the range of values reported for other gamma models in the literature (Table 3). It is reported that *Listeria* has a typical T_min_ value between 0 and −5 °C in meat products [31,49]. A T_min_ value ranging from 0 to −2.83 °C is considered a realistic estimate; while T_min_ of −5.8 °C or below could result in an overestimation of the growth rate, particularly at temperatures between 2 and 5 °C [44]. The estimated T_opt_ of 37.0 °C in the present study is consistent with the typical optimum temperature of 37 °C for *Listeria* growth in meat and poultry products as reported in previous studies [25,47]. The confidence interval for T_opt_ was slightly larger because of the lack of growth data above the temperature optimum region. The estimated pH_min_ of 4.19, aw_min_ of 0.932, and aw_opt_ of 0.998 were consistent with the literature ranges as summarized in Table 3. The slight difference in estimated pH_min_, aw_opt_, and a_wmin_ values compared to previous studies (literature) was mainly due to strain differences. The large confidence intervals obtained for pH_min_ were due to the insufficient growth rate data near the pH_min_ region. The simultaneous modeling technique has no role in yielding a large confidence interval, as reported earlier [28]. In this study, the predicted *Listeria* growth rate was largely dependent on T_min_ and pH_min_, which are independent of other growth conditions. This is evident when the growth rate contour plot (Figure 3) was built with temperature and pH as independent variables using Equation (6), for which a no-growth region is highlighted with dark shades. The growth and no growth interface are consistent with previous studies [24].

#### 3.1.2. Estimation of MIC_s_ of Inhibitory Compounds

The inhibitory effect of organic acids is associated with their ability to reduce the water activity of the food matrix and intracellular pH within the bacterial cells. The organic acid existing in the water phase in its undissociated form is most likely to inhibit *Listeria* growth in the food matrices. This is because the undissociated acid penetrates the cell membrane effectively, reduces the intracellular pH, and disrupts microbial cellular metabolism functions [60]. Therefore, the water phase undissociated concentrations (mM) of the acids were calculated and used for modeling. The estimated MICs (mM) of undissociated nitrite, undissociated water phase organic acids, and their confidence intervals are summarized in Table 3. The estimated MIC_U_ of nitrite was 24.82 mM and was close to the value (25 mM) reported earlier [46]. The MIC_U_ values for organic acids are typically strain-specific, varying with growth medium and conditions. In this study, the estimated MIC_U_ was 18.3 mM for acetic acid, 6.8 mM for lactic acid, and 9.8 mM for propionic acid. In this study, the growth rates under acetic and propionic acid conditions varied linearly with the square root of undissociated acid concentrations, whereas for lactic acid, the growth rates varied linearly. A similar relation between growth rates (μ_max_) and organic acids was reported earlier [47]. The MICs of organic acids are within the range reported in the literature value shown in Table 3. In another study [42], the MIC varies between 6.2 to 18.9 mM (acetic), 3.6 to 5.7 mM (lactic), and 4 to 8 mM (propionic acid) for nine different *Listeria* strains, indicating variable sensitivity of strains to organic acids. Similarly, the estimated MICs ranged between 17.8 and 22.8 mM for acetic acid, 6.9 and 9.1 mM for lactic acid, and 7.6 and 9.9 mM for propionic acid in another study [47]. Therefore, the MIC variation in the current study was mostly attributed to strain differences and RTE food matrices to an extent. The large confidence interval for organic acids was attributed to inadequate data in RTE meat products under these acids’ conditions. This was also evident in another study reporting large confidence intervals ranging between 2.46 and 30.2 mM for a MIC value of 16.3 mM (for lactic acid), due to the inadequate growth data under lactate conditions [28].

### 3.2. Validation of the Secondary Growth Model

**Figure 4:**
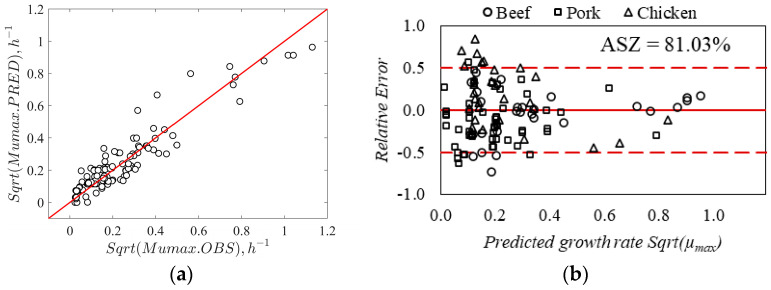
(**a**) Correlation between observed data and simulated growth rates by developed model for *L. monocytogenes*. (**b**) Distribution of relative errors within acceptable simulation zone for the model predictions.

Several *Listeria* models in the literature have been developed in culture media, and limited models have been developed and validated in the RTE meat matrix. It was well established that the growth medium can significantly influence *Listeria* behavior, particularly the complexity of the food matrix [27]. The porosity in food imbalances microbial stability, while starch ingredients can immobilize the cells of bacteria, resulting in retarded growth of cells in food compared to culture media [38,61]. Therefore, the secondary model developed in this study was validated using exclusive RTE meat matrices data collected from the literature (refer to Table 1, Table 2 and Table 3). The estimated gamma model’s parameters corresponding to *L. monocytogenes* (shown in Table 3) using Equations (6)–(12) were reused. The model’s prediction was evaluated by comparing the relative errors between observed and predicted growth rates using the acceptable simulation zone (ASZ, ±0.5 log CFU/g/h) approach. The correlation between the square root of observed and predicted growth rates is presented in Figure 4a; the root mean square error (RMSE) criteria was also computed to assess the average prediction error by the model. In the validation step, the model includes an RMSE of 0.076 μ_max_ and a coefficient of determination (R^2^) = 0.87. For the model predictions, about 81.03% of relative errors were within the acceptable simulation zone (ASZ), which is above the minimum acceptable value of 70% (Figure 4b). The validation of the new secondary model in the present study provided an acceptable *Listeria* growth prediction.

The model’s performance was also compared with and without an interaction effect. Excluding the interaction, the model overestimated the growth rate by 12% (B*_f_* = 1.12) compared to the model with interaction (B*_f_* = 0.96). The accuracy factor was 1.50 for the model with interaction compared to 1.48 for the model without interaction term, indicating no significant variation in prediction accuracy between the two models. However, the interaction effect is important for the robustness of the model as per previous studies. The B*_f_* (0.96) and A*_f_* (1.5) recorded in this study were good and within the acceptable range as reported earlier [58]. For the *Listeria* growth model, a B*_f_* of 0.97 for the model with interaction and 1.31 for the model without interaction was reported earlier [43]. This study also reported that inaccurate predictions always include both fail-safe and fail-dangerous predictions. However, when an equal proportion of over- and under-estimated growth rates tend to “cancel out”, results in an acceptable bias factor close to one. The A*_f_* and B*_f_* values were estimated by replacing the zero-growth predicted (*n* = 2 at 4 °C) values by 0.0003 h^−1^, which demonstrated a significant effect on validation criteria (A*_f_* value). For example, excluding zero predicted μ_max_, the estimated A*_f_* and B*_f_* values were 1.39 and 1.03, respectively. This demonstrates that these factors are extremely sensitive to small deviations in a few predicted growth rate values, resulting in poor validation criteria, though overall model predictions were good. It is not recommended to exclude fail-dangerous predictions by the model; therefore, the current study replaced the zero predicted values with a small growth rate of 0.0003 h^−1^ considering an average shelf life of 4.5 months. The percent bias (%B) of 12.7% for the model without interaction was reduced after including the interaction effect (−4.0%). However, the percentage discrepancy (%D) for both models ranged between 48.1 and 50.4% and did not vary significantly (Table 4). A percent discrepancy of 24.54% for sequential modeling and 29.03% for simultaneous modeling methods reported earlier [28] is close to the range observed in the present study. Similarly, the percentage discrepancy ranging from 48 to 72% was reported for predictions with and without acids and interaction effects [43]. The AIC values for the model with interaction were slightly smaller than the model without interaction, suggesting that the model predictions with interaction are better than the latter (Table 4). Similarly, it was evident from previous studies that considering the interaction effect between the factors improves the quality of fit and prediction accuracy. In addition, it is noted that with the exclusion of the interaction term, the ASZ score was reduced by 4.3%. The model with interaction produced an ASZ-score of 81.0%, and the incorrect predictions were fail-safe (8.6%) and fail-dangerous (10.3%) predictions. Typically, a model performance is accepted if at least 70% of its predictions fall within the acceptable simulation zone (ASZ). A study [25] first reported a no-interaction model, which had less prediction accuracy; later, the revised with-interaction model significantly reduced the fail-safe (13.5 to 12.1%) and fail-dangerous (16.1 to 7.1%) predictions [24]. Similarly, the inclusion of the interaction effect to model the correct predictions increased from 69 to 89% [62], and 62–81 to 85–87% [29]. Another gamma model without the interaction term overestimated the growth rate by 31–33% [43]. These studies indicate better prediction accuracy for models with interaction terms. In the present study, both the models with and without interaction effect offered an acceptable bias factor (1.12 and 0.96, respectively) and ASZ score (76.7 to 81%, respectively). In a study, the [62] model produced an overall prediction of 65% (ASZ) in broth data and 89% in meat, seafood, poultry, and dairy products [30].

### 3.3. Validation of Predicted Lag Time in RTE Meats

**Figure 5:**
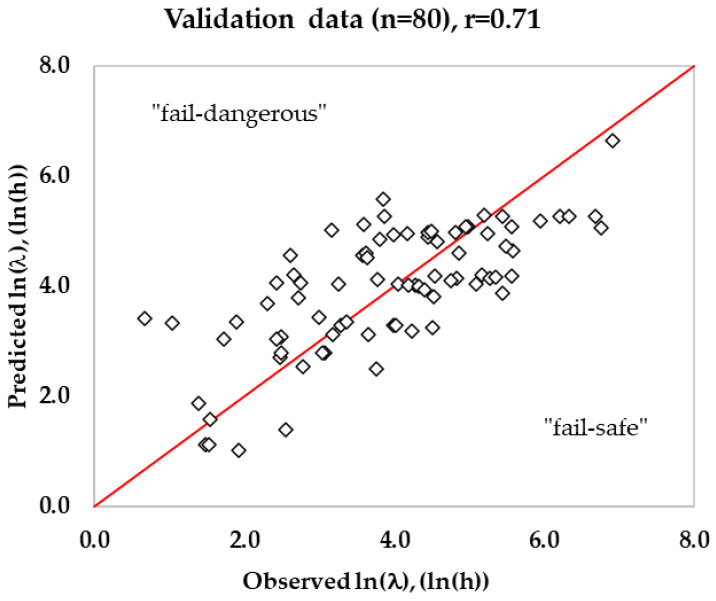
Predicted lag time by the developed gamma model against observed lag time in RTE meats for the validation data set.

A total of 596 growth and no growth data were collected in this study, out of which only 480 growth rate (μ_max_) data and their corresponding lag time (including lag = 0) qualified the first filter criteria (Table 5) to develop a secondary growth rate model. The second filter criteria were applied to exclude zero lag time and select good quality data for lag time modeling. Out of 480 training data, about 310 lag time values were used to estimate the parameter K. Similarly, out of 116 validation lag time values, about 80 lag time values (excluding R^2^ ≤ 0.9, Obs. lag < 1 h, Pred. μ_max_ < 0.0001/h (*n* = 2)) were used to validate the lag time model. The logarithmic ratio of lag and generation time resulted in a median value of 1.31, and the exponential of the median value was estimated to be 3.72 (parameter K). During validation, the lag time was estimated using Equation (25) with the value of growth rate from the growth rate model with interaction and with the previously estimated physiological state of cells (K = 3.72). In a similar study, a median value = 1.128 and K = 3.09 were estimated (*n* = 1176) for *Listeria*, including culture broth, dairy, meats, eggs, and seafood as growth mediums [25]. The K value in the current study for RTE meat products is slightly higher (3.72) than the previous study (3.09), and it may be attributed to K estimated from a mixture of culture broth (shorter lag phase), dairy, and seafood. In this study, the estimated B*_f_* was 1.07 for the lag time model, indicating that the predicted lag time was fail-dangerous by 7% for the validation dataset (Figure 5). The mean prediction error (MPE) for the lag time model estimated using Equation (26) was 137.3% for the training dataset, which was slightly less (128.8%) for the validation dataset. The RMSE value for the predicted lag time was 0.98 ln(h), and the correlation coefficient (r) was 0.71. The lower correlation coefficient indicates that the lag time model is less accurate than the growth rate model.

## 4. Discussion

**Figure 6:**
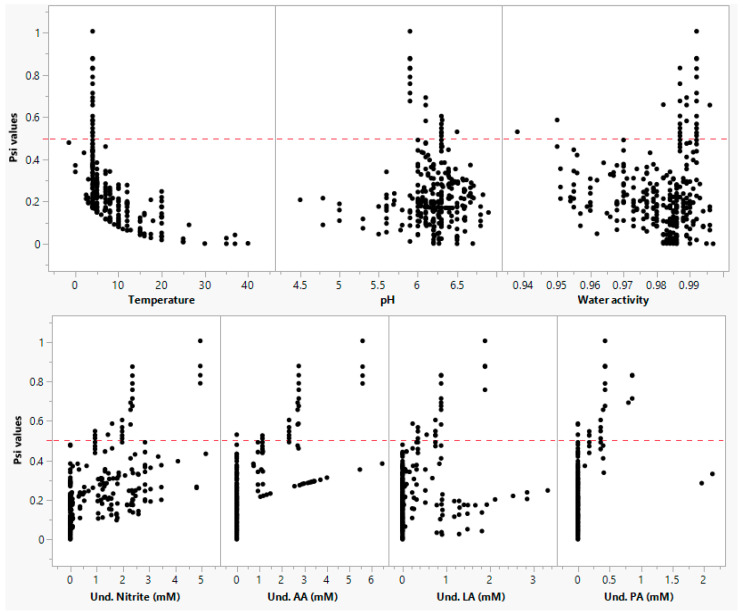
Interaction effects under different environmental conditions. Note: AA, LA, and PA refer to milli molar concentration (mM) of undissociated acetic acid, lactic acid, and propionic acid, respectively.

**Figure 7:**
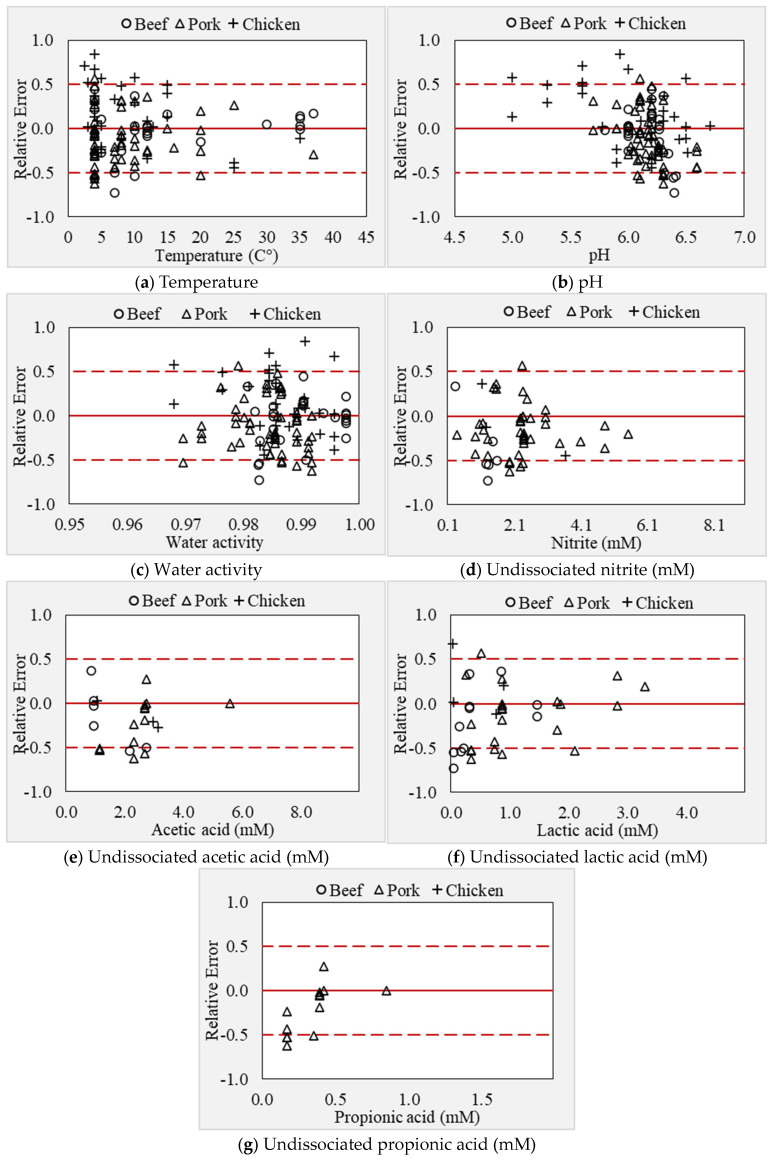
(**a**–**g**) Relative error plots as a function of storage and inhibitory factors.

In the current study, we demonstrated a method to develop and validate an improved secondary model using limited and disparate RTE meat matrix data from the literature. The model may be used to predict the growth of *L. monocytogenes* in ready-to-eat and processed meat and poultry products. To date, several models have been developed using culture media data; very few models are validated in the food matrices, and limited models have been developed and validated in exclusive food matrix data. In this context, this study demonstrated that the “simultaneous” modeling approach is an effective method to develop and validate new models for different pathogens using limited literature data in food matrices. In the validation step, the developed model prediction was evaluated across the different meat products. The validation data included 29 growth rates in beef products, 56 growth rates in pork products, and 31 growth rates in chicken products. The developed model growth rate predictions were fail-dangerous by 9% and 6% in beef and chicken, respectively, and fail-safe by 3% in pork products (Figure 7a–g). The under-prediction in beef meat was associated with the presence of multiple organic acids and nitrite in the product formulation, and most of these data were within the temperature range of 4 to 10 °C. The overestimation of the synergistic effect between multiple factors (low temperature and antimicrobials) resulted in the fail-dangerous predictions by the model. In contrast, the failure-dangerous prediction in chicken products was because the model underpredicted the growth rates at low pH (<5.6) conditions. The RMSE for beef products was found to be the highest (0.091, μ_max_) compared to beef (0.079, μ_max_) and pork (0.059, μ_max_) products. The interaction effect between environmental factors, as described by Psi (ψ) values, is shown in Figure 6. About 9.2% of Psi (ψ) values ranged between 0.5 and 1, indicating the interaction effect between the factors. The interaction effect was almost zero when the temperature was close to optimum, whereas it exponentially increased with a decrease in temperature. These observations indicate that the interaction between temperature and organic acids was significant. Particularly, the highest interaction effect was recorded under low-temperature conditions including a combination of multiple microbial inhibitors. The effect of antimicrobials and their synergistic effects may slightly subside when the temperature of growing conditions increases close to optimum value. However, the effect of temperature on the inhibitory potential of antimicrobials and the interaction between these factors could not be comprehensively assessed in high-temperature regions (20 to 37 °C) due to the lack of antimicrobial data at higher temperature conditions.

During modeling, where there is limited data to estimate certain parameters, it is recommended to fix these cardinal parameters without estimating them. This approach can reduce variation, as suggested by [28]. In many studies, the T_opt_ value was fixed at 37 °C during the fitting procedure, when the available growth data are limited above the temperature optimum. However, this is inappropriate when adequate data exists because it may result in over- or under-estimation of the growth rate. This is because a fixed T_opt_ value may impact the smoothness of fit (skewed fit), leading to inaccurate growth rate prediction, as evident in this study. However, when modeling a single strain, the strain-specific parameters (T_opt_) may be fixed, as demonstrated by [32]. For example, in their study, T_opt_ was fixed at 38.9 °C for *L. monocytogenes* ADQP105, and other parameters were estimated. Due to the lack of sufficient data for temperatures above the optimum range, the model’s applicability in some storage and processing scenarios is limited. Nevertheless, the cardinal parameters estimated in the present study are consistent with the literature values. In this study, the model slightly underestimated the growth rate at temperatures between 30 °C and 37 °C (*n* = 3) and −1.5 °C (*n* = 1). Conversely, at 4 °C, the model predicted zero growth in two conditions, including multiple antimicrobials (2.2 to 4.9 mM nitrite, 2.69 to 5.58 mM acetate, 0.88 to 1.88 mM lactate, and 0.39 to 0.42 mM propionate) in RTE ham [81]. This was because of the overestimation of interaction effects between factors by the model. In contrast, the model predictions were fail-safe, particularly at the lower temperature range between 4 and 6 °C due to the synergistic effect of multiple organic acids under these conditions (Figure 7a).

The MICs of undissociated nitrite and organic acids estimated in this study slightly varied from literature values (Table 3), and this could be due to variability in strains, food matrices, endogenous inhibitors (e.g., natural lactic acid), and their interactions with added acids, acid calculation method, modeling techniques, and experimental methods used [27]. For example, the MICs of lactic acid ranged from 3.6 to 5.7 mM across nine strains, MICs of acetic acid ranged from 6.2 to 18.9 mM across four strains, and MICs of propionic acid ranged from 4 to 8 mM across three strains [42]. Previous studies have reported a significant difference in growth parameters between strains of *L. monocytogenes* [43,82]. It is important to mention that the MIC estimated in culture media would be different from the MIC estimated in the meat matrix, including background microflora and growth inhibitors. A study by [83] recorded that the *Listeria* growth rate was different under different food matrices, and the exponential growth rate varied significantly among twenty *Listeria* strains. Therefore, establishing a universal MIC value for organic acids is challenging due to intra-species variation. Another study [48] reported the dependency of MICs of undissociated acids on pH and a significant variation in MIC_s_ of organic acids between the *Listeria* strains. This study also indicated that strain variation significantly influences the concentration of acids required to inhibit growth. For instance, in their study, the MIC_s_ for different strains for the pH range studied varied between 12.2 and 30.2 mM for acetic acid and 4.7 and 25.1 mM for propionic acid. The optimized MICs in the current study could be a universal MIC value for all inhibitory compounds in different meat products, including added acids and endogenous lactic acid. In addition, the wide confidence intervals for the MICs of inhibitory compounds could be either due to *Listeria* strain differences or limited data points in particular regions [42,48,84]. In the current study, the large confidence intervals of MIC of antimicrobials and curing agents were attributed to a lack of growth data, which is one of the limitations of this study. In addition, another study [48] reported that limited (2 to 46 data points per acid) MICs of undissociated acids for *L. monocytogenes* are available in the literature; such large intervals of undissociated acid values may result in the approximate estimation of the minimal acid concentration that prevents *Listeria* growth. Therefore, for accurate estimation of the MICs of antimicrobials, it is important to have adequate datasets with closer intervals of undissociated acids. In addition, the undissociated acid concentrations were calculated in this study based on the total volume of food (meat), while acid dissociates in the water phase of unknown volume. The concentration of acids in the water phase is more than the concentration in total meat, which certainly explains the differences in MICs estimated in different studies [27]. Another reason for MIC variations is the differences in the growth substrate, as most literature MICs were estimated in culture media, while the present study estimated MIC in meat matrix.

In this study, the model prediction was impacted by the presence of additional antimicrobial or additive compounds in meat formulations that were not considered in the modeling. For instance, in a few studies, the product formulation included one or more compounds such as sodium erythrobate, phosphate, sodium bicarbonate, sodium triphosphate, sodium ascorbate, and other seasoning blends [67,85,86,87,88,89]. Similarly, RTE products such as ham, frankfurters, smoked meat, and sausages included phenols generated from smoke components. These additives may have an indirect inhibitory effect; a study [49] demonstrated the effect of phenols (>10 ppm) in smoked meats on *Listeria* growth and on A*_f_* and B*_f_* values. Similarly, in the present study, the model overpredicted growth rates in a few products containing the above additives. Therefore, to improve the accuracy of the model’s predictions, it is crucial to consider all the factors that have the potential to inhibit the growth of *Listeria*. At present, there is no single model that considers all factors that may be relevant to all types of food. Therefore, it is important to clearly state the limitations of predictive models to users, including the range of conditions, interpolation region, and the variables considered.

The validation of models in food matrices is important in predictive microbiology as it builds confidence for acceptance. Undoubtedly, *listeria* growth in culture media differs from food matrix as growth is slower in the latter. Model validation in culture media often overestimates the growth in the food matrix due to the complexity of food structure affecting the spatial distribution of bacteria [37]. In the present study, the model was validated in food matrices (*n* = 116) which include ready-to-eat products of beef, pork, and poultry (Table 5, Table 6 and Table 7). The developed secondary model can quantify *Listeria* growth as observed in naturally contaminated RTE food as a function of seven factors studied. The model is useful to simulate the inhibitory effect of antimicrobials and predict the growth of *Listeria* in the presence of nitrite, organic acids, or a combination of nitrite and organic acids in ready-to-eat meat products. The current study presents an improved model that is considered to predict the *listeria* growth that is observed in naturally contaminated food products. A model developed and validated in RTE meats may offer better prediction than the broth and generalized foods model. In comparison to the broth model, the meat matrix models may assist in avoiding overprocessing of food, significantly reducing the ingoing and cost of antimicrobials and reducing potential health risks of antimicrobials. The model may be helpful to food manufacturers to reduce the number of challenge studies, formulate or reformulate, and assess the microbial safety of food.

Based on the correlation coefficient, the developed lag time model was less accurate than the growth rate model in this study. To measure the accuracy of the lag time model, the mean and median errors were estimated following the method reported earlier [25]. The % mean error of the lag time model was found to be 128.8% and was slightly less than the 133% reported earlier [25]. If the observed lag phase was close to zero while the predicted lag was a small lag phase, the % mean error was extremely high, even though the actual error was relatively small. If the predicted lag phase is large, then the % mean error would be immensely high. Due to this reason, the median error was calculated, which would be a better representation. The % median error for the lag time was also still higher, 61.1% (validation dataset), indicating that the prediction of lag time is a big challenge. The estimated mean and median error in this study was close to 133% and 62% reported earlier [25] for lag prediction in a mixture of culture broth and RTE foods (eggs, dairy, seafood, and meat products). The B*_f_* of 1.07 indicated that the predicted lag time was 7% biased in the current study compared to the 3% bias reported in the above study. The experimental variability and the unpredictable *Listeria* behavior result in lag phase variability, which is higher than that for the growth rate. The lag phase occurs at the extreme end of the microbial growth curves and therefore is highly susceptible to experimental error. In addition, large growth rate data are used to develop a valid model, while a limited number of lag data are generally available for lag time modeling. The longer lag phases are observed under extreme stress conditions such as low temperature and high concentration of antimicrobials, where the bacterial cells experience maximum stress, resulting in high variability. For example, a study reported a 0 h lag time at 5 °C, while at 8 °C a higher lag time of 231 h was recorded in comminuted beef [63]. Similarly, another study on cooked pork ham reported a lag time of 0 h at 4 °C and 94 h at 10 °C [90]. Due to this reason, the accuracy of the lag time model for *Listeria* will be poor. For example, an A*_f_* of 2.23 was recorded in the current study for the lag time prediction in validation data. The high variability of lag resulted in a large spread in the scatter plot (Figure 5) between ln(l) vs. ln(Tg.) and a large distribution of the ratio ln(l/Tg). This suggests that the initial assumption of negligible effect of stress conditions on the ratio for cells having similar preincubation history, may not be accurate. This is because there is sufficient evidence in the literature that the ratio ln(l/Tg.) decreases as temperature, pH, and a_w_ approach the optimum values, indicating that the amount of work to be done may be reduced as growth conditions approach optimal [25,91]. The lag time estimated by the model under extreme stress conditions was less accurate compared to mild stress under optimal conditions. Similarly, pre-incubation temperature is also crucial, because a lower pre-incubation temperature could shorten the lag phase duration at low temperatures, while a higher pre-incubation temperature could extend the lag phase duration [92]. Furthermore, the initial inoculum levels, although they do not affect significantly on growth rate, affect the lag phase durations. The low inoculum levels would produce longer lag phases compared to shorter lag phases under high inoculum levels [93,94]. Another reason for the variability in lag time may be because of the strain difference that contributed to the variation in the ratio of lag and generation time [95]. Therefore, it is observed that accurate prediction of the lag phase is more challenging than growth rates. Due to this reason, there are limited studies published on the lag time model in the literature. Therefore, a combination of large datasets (lag time) along with advanced predictive modeling approaches, such as AI models that account for environment and physiological variability, seems to be promising for achieving better accuracy in lag time modeling.

**Table 6:** *Listeria* growth data in pork products, product characteristics, and storage conditions.

Products	No. of Strains	*n*	T (°C)	pH	NaCl	a_w_	Acetate (%)	Lactate (%)	Propionate (%)	Nitrite (ppm)	Data Source	Reference
Wiener pork and Bratwurst	5	4	3–7	5.9–6.3	1.5–2	0.97–0.98		1–6		156	Pub	[14]
Ground cooked ham	5	36	4–10	5.7–6.1	2.4	0.986		1–2		1	Cb	[90]
Cooked Pork liver sausage	1	24	5–20	6.01–6.12	2–4	0.97–0.98		0–4		**98.5**	Pub	[96]
Sliced cooked ham	6	9	7	6.22	3	0.978		0–3			Cb	[97]
RTE Products	5	4	4	**6.13–6.2**	2	0.983–0.99		0–2.5		97	Pub	[87]
Cooked Cured sliced ham	3	18	4–12	6.2	2.8	0.979		0–2		190	Pub	[86]
Ham	8	3	4	6.27–6.42	**2.2**	0.984		0.108–0.12			Pub	[65]
Pork-beef frankfurter	3	18	4–12	6.02–6.17	2.2	0.979		0.2		156	Pub	[98]
Pork-beef bologna	5	1	4	6.07–6.14	2.13–2.16	0.979–0.98			0–0.05	156	Pub	[88]
Cooked ham and Mortadella	1	12	4–12	6.1–6.3	2.5–2.8	0.976–0.979		0.43–0.7		102	Pub	[31]
Pork Bologna	10	4	4–10	6.3–6.5	2	0.98		0–1.8		156	Pub	[99]
Servelat sausage and cooked ham	3	4	4–9	6–6.3	2–2.5	0.98		0–2.5			Pub	[100]
Sliced Cooked Ham	5	5	4	6.39	2.59	0.967		1.6		156	Pub	[101]
RTE ham model	5	90	4	5.5–6.6	0.5–2.5	0.98–0.99	0–0.74	0–3.06	0.05–0.3	0–200	Pub	[81]
Sliced cooked Cured ham	3	8	5–10	6.2	**2**	0.985			0-0.2	**98.5**	Pub	[102]
Cooked sliced ham	3	5	8	6.2	3	0.978					Cb	[103]
Pack Slice Cook Pork	1	2	4	5.99–6.05	2	0.985					Cb	[104]
Ham	1	14	0–15	6.1–6.4	2.2–2.4	0.982–0.984				84–110	Cb	[105]
Pork ham	1	8	0–15	6.6	2.7–4	0.97–0.98				11–170	Cb	[73]
Ham	1	10	47.2	6.4	**2**	0.985					Cb	[106]
Mortadella (bologna ham)	2	10	8	6.19	2	0.981				60	Cb	[107]
Cooked cured pork shoulder	1	12	0–16	6.26	**2**	0.985				150	Cb	[108]
RTE ham and sausages	3	8	4–35	**6.2**	**1.8–2.2**	0.984–0.986					Pub	[109]
Cooked Cured Pork Sausage	5	30	7	6.59	1.84	0.985				50–300	Pub	[110]
Cooked ham	1	2	7	6.1–6.2	**2.2**	0.984					Cb	[71]
Pork Live pate	5	16	4–10	6–6.15	1–3	0973–0.991				0–200	Pub	[111]
Sliced Cooked ham	1	5	2–15	6.07	2.72	0.98				100	Pub	[85]
Processed meats- bologna	5	7	4.4	4.8–6.3	2.3–3	0.95–0.97				0–48	Pub	[79]
Pork Chorizo	1	13	5–30	4.79–6.5	**1.84**	0.984					Pub	[112]
Meatballs and Sundae	7	10	5–37	5.6–6.9	0.5–2.1	0.997–0.989					Pub	[113]

Search criteria in ComBase: beef, poultry, turkey, chicken, pork, RTE meat products, except seafood, *Listeria*, static condition. n is the number of growth curves. Bold type: assumed values. The assumed nitrite value is the average nitrite concentration reported in other products. Data source: Pub—publications, Cb—ComBase, PC—personal communication.

**Table 7:** *Listeria* growth data in poultry products, product characteristics, and storage conditions.

Products	No. of Strains	*n*	T (°C)	pH	NaCl (%)	a_w_	Acetate (%)	Lactate (%)	Propionate (%)	Nitrite (ppm)	Data Source	Reference
Turkey slurry	4	6	4–25	5.2–6.2	1.3–2.1	0.98–0.99		0–2.5		200	Pub	[114]
RTE sliced turkey breast	3	18	4–12	**6.2**	2.2	0.98		0–2			Pub	[86]
Uncured Turkey	8	7	4	6.1–6.4	**2**	0.98				**98.5**	Pub	[65]
Uncured turkey	5	1	4	6.19	**2**	0.98			0.05		Pub	[101]
Comminuted Chicken	3	9	5–35	6.5	**2**	0.98		0–4			Pub	[66]
Chicken salad	5	5	4–12.8	5.6–6	0.61	0.99		0.045–0.051			Cb	[115]
Sliced cooked Turkey bologna	7	3	4	6.5–6.7	2	0.98	0–0.5	0–2		156	Pub	[116]
Sliced Cooked Ham	5	5	4	6.42	1.7	0.972		3.2	0.05–0.3		Pub	[88]
Cured Deli Style Turkey	5	8	4–7	6.1–6.4	1.7–1.8	0.97		1.8	0.2–0.5		Pub	[89]
Turkey bologna	5	2	4–7	6.17	2.2	0.973		1.6		60	Pub	[117]
RTE turkey meat	1	2	10	**6.2**	2.5	0.98					Pub	[118]
Sliced roasted turkey	3	3	5–10	6.2	**2.5**	0.98					Pub	[102]
Chicken liver pate or minced chicken breast	1	2	6.8–30.4	5.6	0.8	0.99					Cb	[119]
Precooked chicken nuggets	1	3	3–11	6.5	1.5	0.98					Pub	[120]
Chicken breast	2	30	0–15	5.6	2–4	0.97–0.98					Cb	[76]
Dark-meat chicken nuggets	1	3	3–11	6.5	1.5	0.98					Cb	[121]
Sliced Chicken Breast	1	2	7	6.2	**2.8**	0.97					Cb	[71]
RTE chicken salad	3	9	4–16	5.93	**1.3**	0.99					Pub	[122]
Chicken nuggets	6	5	4–16	6.21	**1.5**	0.98					Pub	[123]
Chicken salad	3	9	5–25	5.9	**0.612**	0.99					Pub	[124]
Processed meats- Sliced chicken	5	3	4.4	6.3–6.5	1.7–2.7	0.97–0.98					Pub	[79]
Cooked deli turkey breast	5	31	5	6.1–6.83	0.6–2.5	0.996–0.982	0–1.35				Pub/PC	[125]

Search criteria in ComBase: beef, poultry, turkey, chicken, pork, RTE meat products, except seafood, *Listeria*, static condition. n is the number of growth curves. Bold type: assumed values. The assumed nitrite value is the average nitrite concentration reported in other products. Data source: Pub—publications, Cb—ComBase, PC—personal communication.

## 5. Conclusions

The current study was conducted to develop and validate an improved prediction model to describe the effects of pH, temperature, a_w_, nitrite, and organic acids on the growth rate of *Listeria* in RTE meat matrices. The gamma model was used to quantify the behavior of *listeria* in ready-to-eat products. It was evident from the present study that better modeling and parameter estimation can be achieved through a simultaneous modeling approach. This approach would enable modelers to develop more robust models on limited and disparate data sets. The current study presents an improved *Listeria* growth prediction model developed and validated in food matrices data. The model performance included B_f_ = 0.96, A_f_ = 1.5, and RMSE = 0.06 μ_max_, indicating the robustness of the model’s prediction. The developed model could offer predictions close to natural growth in meat products. The lag time model compared to the growth rate model is less accurate for lag time prediction. The growth rate model may find its application for microbiological food safety assessment and may be incorporated into a predictive toolbox for research and development purposes. One of the advantages of gamma models is that the impact of a change in the level of a single variable can be calculated without having to reassess the entire equation. This ability to determine the relative effects of different changes could be valuable in risk assessment, particularly when conducting sensitivity analyses aimed at assessing potential strategies for risk analysis.

## Figures and Tables

**Table 1:** Criteria used to filter and select growth rate data for modeling.

Factors	Criteria to Exclude
Growth curves	Curves having less than three points in the exponential phase
Treatments	Irradiation and high-pressure processed samples
Growth rate	The estimated growth rate (μ_max_) equal to zero
Antimicrobials	Products with surface treatment (only products with antimicrobials incorporated into the food formulation were considered)
Poor data	Curves with poor model fitting (R^2^ < 0.9)
Storage atmosphere	Modified Atmospheric Package or CO_2_.

**Table 2:** pKa values and initial guess values of MICs for undissociated organic acid and nitrite.

	Acetic	Lactic	Propionic	Nitrite	References
pKa	4.76	3.8	4.87	3.37	[46,47]
MIC_U_ (mM)	20.3	8.0	8.8	25	

MIC_U_ is the minimum inhibition concentration of undissociated organic acids and nitrite (mM).

**Table 3:** Estimated model parameters in this study with their 95% confidence intervals.

Parameters	Estimated Value	95% CI Values		Literature Range	References
		LCI	UCI		
µ_opt_ (h^−1^)	1.126	0.65	1.66	0.85 to 1.33	[27,43]
T_opt_ (°C)	37.0	34.83	39.38	35.9 to 39.7	[23,43]
T_min_ (°C)	−1.57	−2.14	−1.0	−4.5 to 1.16	[43,47]
pH_min_	4.19	3.56	4.79	4.03 to 4.57	[29]
aw_min_	0.932	0.904	0.938	0.92 to 0.93	[43]
aw_opt_	0.998	0.995	1.00	0.997 to 1.0	[25]
MIC_U_ NIT	22.12	2.36	41.8	11.4 to 25	[27,43,47,48]
MIC_U_ AA	18.33	0.98	37.6	17.8 to 22.8	
MIC_U_ LA	6.88	4.04	9.73	1.7 to 9.8	
MIC_U_ PA	9.88	−9.0	28.8	7.6 to 9.9	

Note: pH_opt_ and pH_max_ values were fixed at 7.0 and 9.6, respectively. NIT is nitrite, AA is acetic acid, LA is lactic acid, and PA is propionic acid in undissociated form. Literature range refers to the lowest and highest values reported in literature.

**Table 4:** Comparison of the model performance with and without interaction.

Model	B*_f_*	A*_f_*	ASZ (%)	%B	%D	FS (%)	FD (%)	AIC
Without interaction	1.12	1.48	76.7	12.7	48.1	15.5	7.7	−228.7
With interaction	0.96	1.50	81.0	−4.0	50.4	8.6	10.3	−230.7

**Table 5:** *Listeria* growth data in beef products, product characteristics, and storage conditions.

Products	No. of Strains	*n*	T (°C)	pH	NaCl	a_w_	Acetate (%)	Lactate (%)	Propionate (%)	Nitrite (ppm)	Data Source	Reference
Comminuted beef emulsion	1	11	5–10	6.30	**2.00**	0.986	0.1–0.2	1.8–2.5			Cb	[63]
Beef Bologna	5	8	5–10	5.9–6.3	**2.50**	0.973		2.50			Cb	[64]
Roasted beef	4	18	4–12	5.80	0.30	0.998	0–0.1	0.2–0.4		**98.5**	Pub	[21]
Roasted beef	8	3	4	5.6–6.19	**1.15**	0.990		0.12			Pub	[65]
Frankfurters	6	21	4–10	6.15–6.4	1.80	0.983	0.25–0.8	0.14–0.25		112.5	Pub	[16]
Comminuted meat	3	25	5–35	6.27	**2.00**	0.985		0–4		140	Pub	[66]
Frankfurter sausage	1	2	4	6.1–6.3	2.04–2.11	0.981	0.12–0.18	0.66–2.26		11–19	Pub	[67]
Frankfurter	5	10	4–10	5.68–6.18	1.73–2	0.980–0.983		0–3		4.1–4.8	Pub	[68]
Frankfurter	1	3	4	**6.20**	**1.80**	0.982		0–2		**98.5**	Pub	[69]
Beef gravy	2	4	5–10	6.00	**1.00**	0.994					Pub	[70]
Luncheon meat	1	2	7	6–6.3	**1.10**	0.992					Pub	[71]
Frankfurter	4	5	15–40	**6.30**	**1.80**	0.982					Pub	[72]
Corned beef	1	5	0–15	6.20	3.25	0.973				5.00	Cb	[73]
Cooked beef	2	4	5–10	5.80	1.00	0.996					Cb	[74]
Sliced roast meat	2	4	−1.5–3	6.10	**1.15**	0.990					Cb	[75]
Beef sirloin	1	5	0–15	6.00	1.80	0.982					Cb	[76]
Frankfurter	1	3	4–18	**6.20**	**1.80**	0.982				98.50	Pub	[77]
Ready-to-eat braised beef	4	24	4–40	6.20	1.00	0.990					PC	[78]
Ham and roasted meat	5	4	4.4	5.4–6.4	0.6–3	0.976–0.992				28–42	Pub	[79]
Ham	1	12	4–7	6.67	1.9	0.986				85	Pub	[80]

Search criteria in ComBase: *Listeria*, a static condition in beef, poultry, turkey, chicken, pork, or RTE meat products, except seafood. *n* is the number of growth curves; Bold type: assumed values. The assumed nitrite value is the average nitrite concentration reported in other products. Data source: Pub—publications, Cb—ComBase, PC—personal communication.

## Data Availability

The original contributions presented in the study are included in the article, further inquiries can be directed to the corresponding author.
